# Host and non-host roots in rice: cellular and molecular approaches reveal differential responses to arbuscular mycorrhizal fungi

**DOI:** 10.3389/fpls.2015.00636

**Published:** 2015-08-13

**Authors:** Valentina Fiorilli, Marta Vallino, Chiara Biselli, Antonella Faccio, Paolo Bagnaresi, Paola Bonfante

**Affiliations:** ^1^Department of Life Sciences and System Biology, University of TurinTurin, Italy; ^2^Institute for Sustainable Plant Protection–National Research CouncilTurin, Italy; ^3^Genomics Research Centre - Consiglio per la Ricerca e la Sperimentazione in AgricolturaFiorenzuola d'Arda, Italy

**Keywords:** arbuscular mycorrhizal symbiosis, *Oryza sativa*, RNA-seq, root apparatus, lateral root

## Abstract

*Oryza sativa*, a model plant for Arbuscular Mycorrhizal (AM) symbiosis, has both host and non-host roots. Large lateral (LLR) and fine lateral (FLR) roots display opposite responses: LLR support AM colonization, but FLR do not. Our research aimed to study the molecular, morphological and physiological aspects related to the non-host behavior of FLR. RNA-seq analysis revealed that LLR and FLR displayed divergent expression profiles, including changes in many metabolic pathways. Compared with LLR, FLR showed down-regulation of genes instrumental for AM establishment and gibberellin signaling, and a higher expression of nutrient transporters. Consistent with the transcriptomic data, FLR had higher phosphorus content. Light and electron microscopy demonstrated that, surprisingly, in the Selenio cultivar, FLR have a two-layered cortex, which is theoretically compatible with AM colonization. According to RNA-seq, a gibberellin inhibitor treatment increased anticlinal divisions leading to a higher number of cortex cells in FLR. We propose that some of the differentially regulated genes that lead to the anatomical and physiological properties of the two root types also function as genetic factors regulating fungal colonization. The rice root apparatus offers a unique tool to study AM symbiosis, allowing direct comparisons of host and non-host roots in the same individual plant.

## Introduction

One of the most important biological novelties that evolved in plant colonization of land was the root apparatus, an organ specialized to anchor the plant body, and to absorb and store water and nutrients. Current knowledge indicates that the ancient alliance between non-rooted plants and symbiotic fungi, such as Glomeromycota and Mucoromycotina, promoted this morphological innovation and has played a key role in the origin of land flora (Brundrett, 2002; Bonfante and Genre, 2008; Gutjahr and Paszkowski, 2013 and citations therein). This ancient alliance continues with most modern plants, as approximately 80% of vascular plant species establish arbuscular mycorrhizal (AM) symbiosis with fungi of the Glomeromycota (Redecker et al., 2013). However, several angiosperm species belonging, for example, to Brassicaceae (including the model plant *Arabidopsis thaliana*), Chenopodiaceae, Cyperaceae, and Proteaceae cannot establish AM symbiosis and are considered non-host plants (Delaux et al., 2013; Lambers and Teste, 2013; Veiga et al., 2013). A recent work on plant-microbe interactions characterized a core set of highly conserved genes required for the establishment of AM symbiosis, the so-called “symbiotic toolkit” (Delaux et al., 2013). Non-host plant genomes lack most (64%) of these symbiotic genes, suggesting that the ancestors of these plant families lost the ability to establish AM symbiosis (Delaux et al., 2013). Among host plants, the eudicots *Medicago truncatula, Lotus japonicus*, and *Solanum lycopersicum* and the monocots *Oryza sativa* and *Zea mays* are considered useful model species to gain insights into the evolution and the mechanisms controlling AM association. Although, eudi- and mono-cotyledonous plants display distinct root system architecture and cellular organization (Hochholdinger and Zimmermann, 2008), both root systems show comparable distributions of AM colonization. In particular, AM fungi preferentially colonize lateral roots and rarely colonize taproots (eudicotyledons) or crown roots (monocotyledons) (Hooker et al., 1992; Gutjahr et al., 2009).

Among AM-host plants, rice (*O. sativa*) has an unusual root system consisting of embryonic and postembryonic crown roots, which branch to generate two types of secondary root: large lateral roots (LLR), which show positive gravitropism and intermediate growth and branching, and the more abundant fine lateral roots (FLR), which do not respond to gravity and never produce lower orders of ramification (Coudert et al., 2010). FLR lack both constitutive and inducible aerenchyma tissues, LLR develop aerenchyma sporadically in dryland and regularly in wetland, and crown roots regularly have aerenchyma, irrespective of water regime (Rebouillat et al., 2009; Vallino et al., 2014). Interestingly, the anatomical differences displayed by the three root types probably mirror a divergent functional role, an issue that has been poorly investigated. To date, it has been proposed that crown roots mainly function to provide anchorage and support, and due to the constitutive presence of aerenchyma, to provide oxygen from shoots to roots, while lateral roots may function to take up nutrients (Kirk, 2003). This hypothesis is supported by a root-type specific transcriptomic analysis performed on CR, LLR, and FLR collected from control and AM colonized root of rice (Gutjahr et al., 2015). CR, in line with their role of plant stabilization, showed an enhanced expression profile of genes involved in secondary cell wall metabolism (SCW), while both lateral roots displayed an enrichment of transcripts related to mineral transport.

AM fungi preferentially colonize LLR, not FLR (Gutjahr et al., 2009; Vallino et al., 2014) but the determinants that make FLR not susceptible to AM fungal colonization remain unknown.

In this work, our investigations were driven by the following hypotheses: (1) LLR and FLR have different gene regulation profiles leading to different developmental plans; (2) the two LR have different functional roles irrespective of the symbiosis; (3) the different anatomy of the two LR is crucial to determining their different mycorrhizal status; (4) multiple factors may determine the different mycorrhizal status. To address these issues, we combined molecular, morphological, and physiological approaches. Through mRNA-seq, we compared LLR vs. FLR in control and mycorrhizal conditions and we focused our attention on candidate transcripts that may: (i) define the differences in anatomy and thus different roles of LLR and FLR, and (ii) make FLR not susceptible to fungal colonization. We generated a comprehensive and integrated data set that provides baseline information for elucidating gene networks associated with root development, functions, and interaction with AM fungi. Finally, we propose the rice root system, with its host and non-host roots present on the same plant, as a powerful system to discover new determinants involved in AM colonization.

## Materials and methods

### Plant material, mycorrhization, growth conditions, and sampling

All experiments were done on *O. sativa* cv. Selenio, a common Italian rice variety with round grains. Seeds (provided by the Rice Research Unit of the Agricultural Research Council, Vercelli, Italy) were germinated in pots containing sand and incubated for 7 days in a growth chamber under 14 h of light (23°C) and 10 h of dark at 21°C. Plants were then transferred individually to new pots in the presence or absence of the mycorrhizal fungus.

*Rhizophagus irregularis* (DAOM 197198), previously identified as *Glomus intraradices* Schenck and Smith (Krüger et al., 2012), was produced in monoxenic cultures maintained on *Agrobacterium rhizogenes*-transformed chicory roots (Bécard and Fortin, 1988) in two-compartment Petri plates, as described in Pérez-Tienda et al. (2011). The extraradical mycelium was harvested from the fungal compartment as described in Vallino et al. (2014).

Mycorrhizal roots were obtained by the sandwich method (Guether et al., 2009). Plants were grown in 9-cm-high and 11-cm-diameter pots and maintained in a growth chamber, as described above, until harvesting (42 days post inoculation–dpi). Plants were watered as described in Vallino et al. (2014). The colonization status of mycorrhizal roots was checked under a microscope.

For RNA-seq experiments, about 30 mg of FLR and LLR were collected manually by using scalpel and forceps to obtain homogenous root sets from both mycorrhizal and control plants (Figure S1). The FLR collected were those originated from LLR, and in the LLR set, the tertiary roots were not included. Collected roots were immediately frozen in liquid nitrogen and kept at −80°C until RNA extraction.

### Nucleic acid extraction

Total genomic DNA was extracted from *R. irregularis* extraradical mycelium and *O. sativa* shoots using the DNeasy Plant Mini Kit (Qiagen, Hilden, Germany), according to the manufacturer's instructions. Plant and fungal genomic DNAs were used to test each primer pair designed for real-time PCR to exclude cross-hybridization.

Total RNA was extracted from rice roots of mycorrhizal and non-mycorrhizal plants using the Plant RNeasy Kit (Qiagen), according to the manufacturer's instructions. Samples were treated with TURBO DNase (Ambion, Austin, TX, USA) according to the manufacturer's instructions. The RNA samples were routinely checked for DNA contamination by RT-PCR (OneStepRT-PCR, Qiagen) analysis, using *OsRubQ1* (Güimil et al., 2005; Table S1).

### IlluminaGAIIx sequencing

Three micrograms of total RNA were used for library preparation with the TruSeq RNA sample preparation Kit (Illumina, FC-122-1001) following the manufacturer's instructions. Libraries were amplified with 15 cycles of PCR and then purified and size-selected to an average size of 300 bp on a 2% low range ultra agarose gel (BIO-RAD).

RNA quality and library concentration and size were assayed on a 2100 Bioanalyzer (Agilent). Libraries were single-end sequenced (51 bp; two samples per lane) on Illumina Genome Analyser (GAIIx). Three biological replicates were generated for each condition, but only two replicates produced good RNA for FLRmyc. Therefore, for FLRmyc thesis, only two samples were considered, complying with the recommended RNA-seq standards that two biological replicates are sufficient (ENCODE Project, 2011 - http://genome.ucsc.edu/ENCODE/protocols/dataStandards/ENCODE_RNAseq_Standards_V1.0.pdf).

### cDNA synthesis and real-time quantitative RT-PCR

Single-strand cDNA was obtained as described in Vallino et al. (2014). Quantitative Real-Time PCR was used to measure the expression of 12 genes shown to be differentially regulated by RNA-Seq. Three biological replicates were conducted for each condition. Quantitative real time PCR experiments and data analysis were carried out as described in Vallino et al. (2014). The primer names and corresponding sequences are listed in Table S1.

### Mapping of illumina reads

Raw fastQ files were checked for low-quality reads and contaminants. Low-quality reads (quality ≤ 10 phred score) and contaminants were removed with Cutadapt software (Martin, 2011). Contaminant-free, filtered reads were mapped with Bowtie/Tophat version 1.4.1 (Trapnell et al., 2012) to the rice genome (*O. sativa* Nipponbare MSU 6.16 release). A minimum and maximum intron length of 40 and 50,000 bp, respectively, were used. Read counts were collected as described in Bagnaresi et al. (2012).

### DEG calling and go enrichment analyses

The DESeq Bioconductor package version 1.10.1 (Anders and Huber, 2010) was used to call Differentially Expressed Genes (DEG), as described in Zouari et al. (2014). One single DESeq CountDataSet object instance was created for both FLR and LLR and the two treatments. DESeq Parameters for dispersion estimation were: method “pooled” and sharing Mode “fitOnly.” The False Discovery Rate (FDR) threshold for DEG calling was set to 0.05. GO enrichment was done as described in Bagnaresi et al. (2012). The GOSEQ Bioconductor package was used to account for RNA length bias typical of RNA-seq approaches (Oshlack and Wakefield, 2009).

### Miscellaneous bioinformatic techniques

Heatmaps of clustered samples were obtained upon transformation of count data values with VST function as available in DESeq R package (Anders et al., 2015). Mapman figures were generated upon binning of DEG sequences to mapman bins by Mercator application (Lohse et al., 2014). Unless otherwise stated, further graphical outputs were generated with custom R and Python scripts.

### Transmission electron microscopy

Root segments from each independent sample were fixed in 2.5% (v/v) glutaraldehyde in 0.1 M cacodilate buffer (pH 7.2) for 2 h at room temperature and then overnight at 4°C, rinsed twice and post-fixed for 1 h in 1% (w/v) OsO_4_. After rinsing in the same buffer, they were dehydrated in an ethanol series at room temperature, followed by two changes of absolute acetone and then infiltrated in Epon-Araldite resin (Hoch, 1986). The resin was polymerized for 24 h at 60°C. Embedded samples were processed for ultramicrotomy. Semi-thin sections (1 μm) were cut from each sample, stained with 1% toluidine blue and observed under an optical microscope to inspect general morphology. Ultra-thin sections (0.05 μm) were counterstained with uranyl acetate and lead citrate (Reynolds, 1963) and observed under a Philips CM10 transmission electron microscope. Some ultra-thin sections were stained using the Thiéry reaction (Thiéry, 1967) (PATAg-staining) to visualize polysaccharides (Roland and Vian, 1991). PATAg-staining uses the oxidation of polysaccharides by periodic acid, creating aldehyde groups, which are visualized by a silver complex.

### Root staining and drug treatment

Different portions of crown roots, LLR, and FLR were embedded in 8% low melting-point agarose and sectioned with a series 1000 Microtome Sectioning System (Vibratome, St. Louis, MO, USA). Cross sections of 100 μm thickness, were observed under a light microscope [Primo Star Zeiss (Carl Zeiss MicroImaging, Göttingen, Germany) with a Leica DFC425 digital camera (Leica Microsystems, Wetzlar, Germany) attached].

Lignin was detected by staining with 1% (w/v) phloroglucinol in 35% (v/v) HCl. Stained lignin appears red under white light. Suberin and cutin were detected by staining for 2 h with 0.1% (w/v) Sudan Red 7B (Sigma) and then mounting in 75% (v/v) glycerol (Brundrett et al., 1991). Photographs were taken within 60 min of staining.

For cutin/suberin monomer treatment, 28 days old mycorrhizal rice plants, previously colonized by *R. irregularis* by means of the sandwich system, were treated for 4 days in hydroponic condition with sterilized Long Ashton solution (with 32 μM Na_2_HPO_4_·12H_2_O; Hewitt, 1966) containing both C16 cutin/suberin monomers: 16-hydroxyhexadecanoic acid and 1,16-hexadecanediol (20 μg/ml) (Wang et al., 2012). The presence of hyphopodia was monitored by screening 400 FLR for each biological replicate. Treatment with equivalent dilutions of ethanol were used as a control. Three biological replicates were considered for each condition. The colonization level of LLR was assessed according to Trouvelot et al. (1986).

For paclobutrazol (PAC) treatment, a gibberellin acid synthesis inhibitor, seeds were sterilized and germinated 5 days in the dark and 4 days at light on Murashige and Skoog medium plates supplemented with 10 μM PAC. Plants were transferred to pots, and in order to get more FLR, plants were harvested after 21 days of growth. Plants were watered once a week with water and once with water containing 10 μM PAC. The shoot and root phenotype was evaluated macro- and microscopically. The number of cortical cells in both treatments was counted by microscopy of vibratome sections of FLR, obtained as described above.

### Phosphorus quantification

FLR and LLR from non-mycorrhizal and mycorrhizal plants were collected manually as described above, from four independent plants. For phosphorus (P) quantification, about 2 mg of dried material was digested in 1 mL 6 M HNO_3_ for 1 h at 95°C. The analysis was performed as described in Zouari et al. (2014).

### Statistical analysis

For all the RT-qPCR, drug treatment, and phosphorous quantification measurements, values are expressed as mean ± standard deviation. Data were analyzed with a One-Way ANOVA with Tukey *post-hoc*, using a probability level of *P* < 0.05. All statistical elaborations were performed using the PAST statistical package (version 2.16; Hammer et al., 2001).

## Results

RNA was isolated from LLR and FLR of rice plants, grown in the absence (LLRc and FLRc) or the presence (LLRmyc and FLRmyc) of the mycorrhizal fungus *R. irregularis*. In order to obtain a homogenous LLRmyc sample, we selected under a stereomicroscope only LLR exhibiting external mycelium. The mycorrhizal status of this sample was confirmed by the calculation of the total root length colonization (76.3% ± 3.2, consistent among replicates) and by the higher expression of the arbuscular marker gene *OsPT11* than control (Figure S2). FLRmyc sample did not show any fungal structures (data not shown). RNA was subjected to single-end whole transcriptome sequencing, obtaining 13–20 millions (mean 17.5 million) reads (51 bases, single-end; Table S2). An RPKM (Reads per Kilobase per Million) cutoff value of 0.1 was set to declare a locus expressed, resulting in 30,204 loci above the expression cutoff. Pearson correlation coefficients for biological replicate samples sharing the same treatment and tissue were always above 0.9 (Figure S3) indicating a good level of reproducibility among replicates.

### Differentially regulated genes in LLR and FLR: A global view

**Figure 1:**
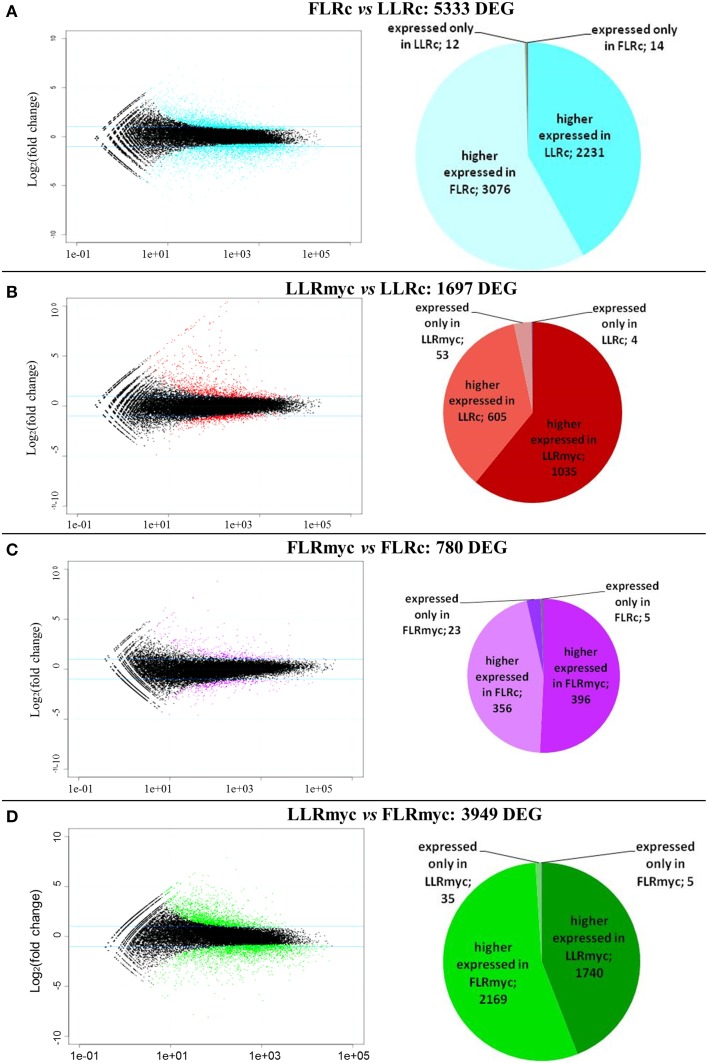
**Global overview of the transcriptional changes in the two root types in presence or absence of the AM fungus**. Mean expression vs. log_2_ fold change plots (MA-plots, left side) were computed for the four comparison: **(A)** LLRc/FLRc, **(B)** LLRmyc/LLRc, **(C)** FLRmyc/FLRc, **(D)** LLRmyc/FLRmyc. Called DEGs (FDR 0.05) are plotted in color. Pie charts (right side) show number of higher-, lower-, specific regulated genes for each condition in each comparison.

We used the R package DESeq to identify DEG among the four tested conditions. Expression values for all genes and comparisons, and annotations of the genes, are reported in Tables S3–S6. The comparisons (Figure 1) included LLRc vs. FLRc (5333 DEG), LLRmyc vs. LLRc (1697 DEG), FLRmyc vs. FLRc (780 DEG), and LLRmyc vs. FLRmyc (3949 DEG). The expression profile of 12 genes randomly selected from those identified in the RNA-seq experiment was successfully validated by qRT-PCR (Figure S2).

**Figure 2:**
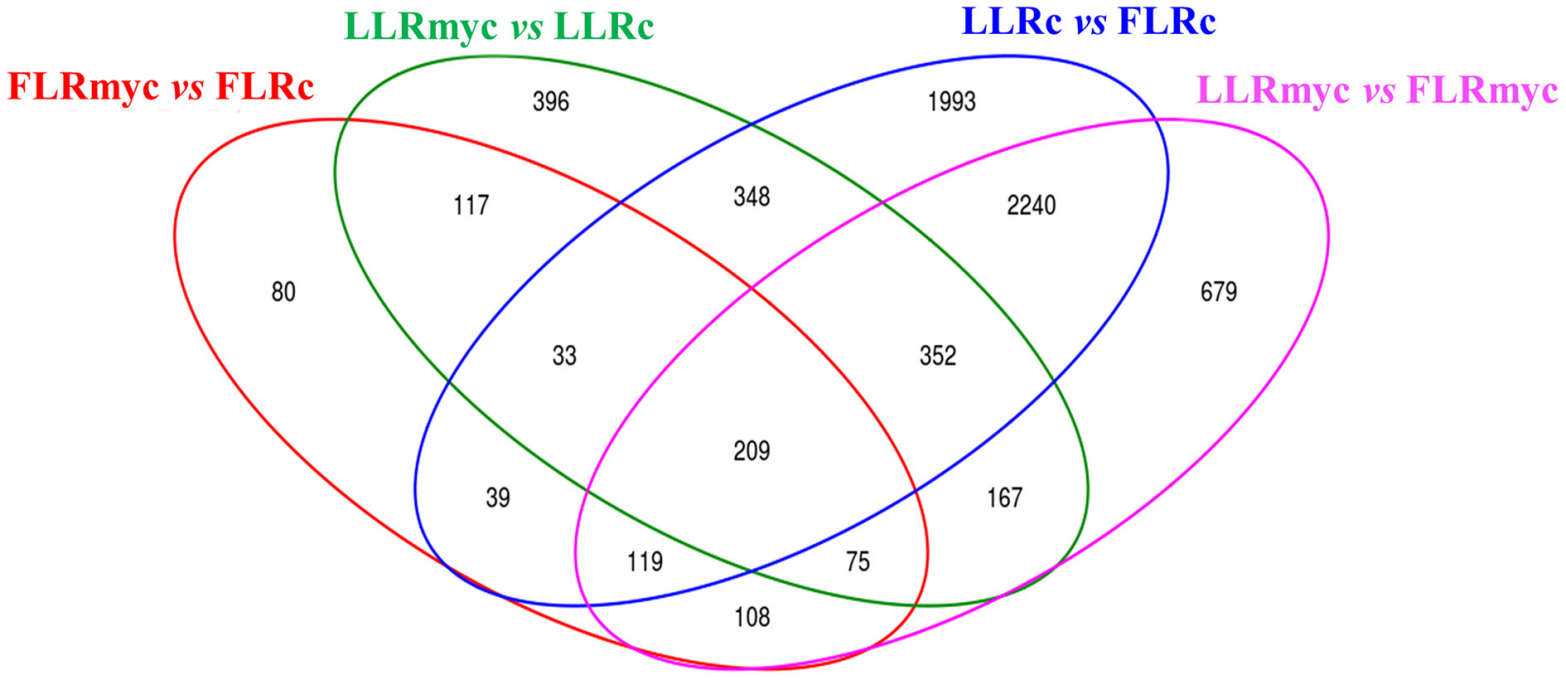
**Venn diagrams of control and AM fungal-modulated genes (DEG)**. Venn diagrams illustrating the relationships between DEGs in contrasts among same tissue and different treatment (myc vs. control) or same treatment and different tissue (LLR vs. FLR).

**Figure 3:**
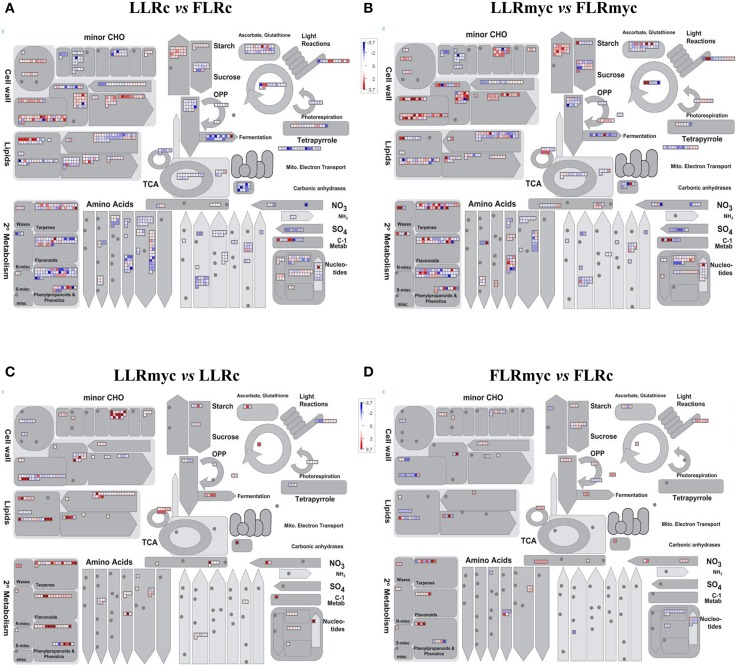
**Overview of the effects on metabolism of the expression changes recorded in the four comparisons: LLRc vs. FLRc (A), LLRmyc vs. FLRmyc (B), LLRmyc vs. LLRc (C), and FLRmyc vs. FLRc (D)**. MapMan software (Metabolism_overview panel) was used to provide a snapshot of modulated genes over the main metabolic pathways. DEGs were binned to MapMan functional categories and log2 fold changes values are represented. Higher- and lower-regulated transcripts are shown in red and blue, respectively.

In all the tested conditions we identified roughly equal numbers of higher or lower accumulating transcripts, with fold-changes between 5 and −5 (log2 scale) and only a few genes going beyond these limits, with exception of the comparison of LLRmyc vs. LLRc (Figure 1). In this comparison, 63% of DEG were up-regulated in the LLRmyc conditions and 62 DEG had a fold-change above 5 (Table S6, Figure 1B), indicating that in this root type, a set of genes (4% of DEG) is strongly up-regulated in response to the presence of the mycorrhizal fungus. The Venn diagrams in Figure 2 showed that 434 genes were differentially expressed (either up or down regulated) both in LLRmyc vs. LLRc and FLRmyc vs. FLRc (25% of the genes modulated in LLR upon infection), and LLRc vs. FLRc compared to LLRmyc vs. FLRmyc had 2920 genes in common (54% of genes modulated in LLRc vs. FLRc). These data suggest that the two roots types are characterized by different transcriptome profiles and the related differences in gene expression are more important than those driven by the presence of AM fungi. DEG data visualized with MapMan software (Thimm et al., 2004) gave the same indication (Figure 3).

In the comparison between LLRmyc and FLRmyc, 35 genes were specifically expressed in LLRmyc and 5 in FLRmyc (Figure 1D). In LLR, which are the preferential host roots for AM fungi, the comparison between the mycorrhizal and the control plants revealed 53 out of 1697 genes specifically expressed upon mycorrhizal colonization. As expected, most of them were already described by Güimil et al. (2005) as AM marker genes, and only three were specifically expressed in LLRc.

We identified the fewest DEG from the comparison between FLRc and FLRmyc, as expected, since the AM fungus does not colonize the FLR. However, the transcript changes suggest that the FLR perceive the fungal presence irrespectively of the fact they are not susceptible to the colonization.

To have an overview of the regulation of the main metabolic and signaling pathways involved in the different comparison, we conducted GO enrichment analysis. Table S7 lists the enriched GO terms for each comparison and Figure S4 shows the GO terms over-represented in both root types in response to AM fungus and the enriched GO terms specific for LLRmyc and for FLRmyc.

A first analysis of the generated data sets largely confirms the first hypothesis that the two roots have their own transcriptome signature.

### Genes involved in AM symbiosis: The comparison between LLRmyc and FLRmyc

To decipher the molecular determinants responsible of the different responses to AM fungus in LLR and FLR, we analyzed the expression profiles of genes described in the literature involved during AM symbiosis. The genes were clustered accordingly to their role in the different stages of mycorrhizal colonization (presymbiotic phase; down-stream Common Symbiotic Signaling Pathway–CSSP; AM marker genes) and are listed in Table 1.

**Table 1:** **List of higher- and lower-regulated genes involved in the different stage of AM symbiosis considering the comparison between: LLRmyc vs. FLRmyc; LLRmyc vs. LLRc; FLRmyc vs. FLRc; LLRc vs. FLRc**.

**MSU LOC_OS ID**	**MSU description**	**LLRmyc vs FLRmyc**.	**LLRmyc vs LLRc**.	**FLRmyc vs FLRc**.	**LLRc vs FLRc**.
**PRESYMBIOTIC PHASE**					
**Strigolactones biosynthesis**					
LOC_Os04g46470	Carotenoid cleavage dioxygenase 7, chloroplast precursor (*Osccd7*)	2.4	2.3	No DEG	No DEG
LOC_Os01g54270	Transposon protein, putative, unclassified (*Osccd8a*) (OsAM180)	3.2	4.1	No DEG	No DEG
LOC_Os09g15240	Carotenoid cleavage dioxygenase, putative, expressed (*Osccd8b*)	3.2	5.7	Ex FLRmyc	Ex LLRc
LOC_Os01g38580	Beta,beta-carotene 9,10-dioxygenase, putative, expressed (*Osccd8c*)	1.6	No DEG	No DEG	1.6
LOC_Os01g50530	Cytochrome P450, putative, expressed	2.3	No DEG	No DEG	1.7
LOC_Os01g50590	Cytochrome P450, putative	2.8	No DEG	No DEG	2.8
**Common symbiosis signaling pathway**					
LOC_Os11g03290	Nucleoside-triphosphatase, putative, expressed (putative *OsLNP*)	4.2	4.5	No DEG	4
LOC_Os03g12450	Nucleoporin, putative, expressed	1.0	No DEG	No DEG	0.9
**Defense response**					
LOC_Os12g36840	Pathogenesis-related Bet v I family protein, putative, expressed	−6.4	1.5	2.7	−5.2
LOC_Os01g25280	Jacalin-like lectin domain containing protein, expressed	−7.8	No DEG	7.2	ND
LOC_Os06g07250	Jacalin-like lectin domain containing protein, expressed	−8.0	No DEG	5.8	No DEG
LOC_Os11g32210	Jacalin-like lectin domain containing protein	−2.0	No DEG	No DEG	No DEG
**LysM-domain**					
LOC_Os01g57400	LysM domain containing protein, putative, expressed—(OsAM3)	3.1	Ex LLRmyc	Ex FLRmyc	ND
LOC_Os01g57390	LysM domain containing protein, putative, expressed (OsAM15)	No DEG	No DEG	No DEG	No DEG
**New receptors**					
LOC_Os04g56360	Cysteine-rich receptor-like protein kinase 8 precursor, putative, expressed	Ex LLRmyc	Ex LLRmyc	ND	ND
LOC_Os09g18530	Serine/threonine-specific receptor protein kinase-like	−6.1	No DEG	No DEG	Ex FLRc
LOC_Os05g25430	receptor-like protein kinase At3g46290 precursor, putative, expressed	ExLLRmyc	0.8	No DEG	3.4
**DOWN-STREAM CSSP**					
**Trascription factors**					
LOC_Os11g31100	Gibberellin response modulator protein, putative (*OsRAM1*)	3.1	6.0	No DEG	No DEG
LOC_Os06g03710	DELLA protein SLR1, putative, expressed	1.1	−1.2	No DEG	1.2
LOC_Os01g67650	Gibberellin response modulator protein, putative (putative *LjRAD1* ortholog)	3.0	Ex LLRmyc	No DEG	ND
**Cutin monomers**					
LOC_Os03g52570	Glycerol-3-phosphate acyltransferase, putative *(OsRAM2*) (OsAM91)	5.0	Ex LLRmyc	No DEG	ND
**AM-MARKER GENES**					
**AM genes Güimil et al., 2005**					
LOC_Os04g04750	Peroxidase precursor, putative, expressed (OsAM1)	2.7	Ex LLRmyc	Ex FLRmyc	ND
LOC_Os05g22300	Transferase family protein, putative (OsAM10)	3.3	7.3	No DEG	No DEG
LOC_Os04g21160	Triacylglycerol lipase 1 precursor, putative (OsAM20)	3.6	Ex LLRmyc	No DEG	ND
LOC_Os06g34470	Zinc finger, C3HC4 type domain containing protein, expressed (OsAM29)	No DEG	No DEG	No DEG ND	
LOC_Os02g03150	Inhibitor I family protein, putative, expressed (OsAM31)	No DEG	No DEG	No DEG	ND
LOC_Os10g18510	UDP-glucoronosyl and UDP-glucosyl transferase domain (OsAM34)	3.3	Ex LLRmyc	No DEG	ND
LOC_Os07g09190	Transketolase, putative, expressed (OsAM38)	2.9	2.6	No DEG	2.5
LOC_Os03g38600	Secretory carrier-associated membrane protein, putative (OsAM42)	4.6	8.4	No DEG	No DEG
LOC_Os09g39520	Cupin domain containing protein (OsAM85)	3.0	5.0	4.3	2.4
LOC_Os12g13170	Transcription factor, putative, expressed (OsAM104)	No DEG	No DEG	No DEG	No DEG
LOC_Os10g33950	Putative uncharacterized protein (OsAM129)				
LOC_Os01g15130	Hydrolase, alpha/beta fold family domain containing protein, expressed (OsAM6)	2.3	Ex LLRmyc	5.6	No DEG
**PT AM-Induced**					
LOC_Os01g46860	Inorganic phosphate transporter, putative (*OsPT11*)	3.4	Ex LLRmyc	Ex FLRmyc	ND
LOC_Os04g10800	Inorganic phosphate transporter, putative (*OsPT13*) (OsAM113)	−1.1	3.0	4.3	No DEG
**AM genes Gutjahr et al., 2009**					
LOC_Os08g34249	Hypothetical protein (OsAM2)	No DEG	No DEG	No DEG	No DEG
LOC_Os06g20120	CND41, chloroplast nucleoid DNA binding protein, putative (OsAM11)	3.0	Ex LLRmyc	Ex FLRmyc ND	
LOC_Os11g26140	Cysteine-rich receptor-like protein kinase 4 precursor, putative (OsAM14)	3.0	Ex LLRmyc	Ex FLRmyc	ND
LOC_Os03g40080	GRAS family transcription factor containing protein, expressed (OsAM18)	2.9	Ex LLRmyc	Ex FLRmyc	ND
LOC_Os02g03190	Inhibitor I family protein, putative (OsAM24)	3.0	Ex LLRmyc	Ex FLRmyc	ND
LOC_Os12g30300	CAMK_CAMK_like.48—CAMK(OsAM26)	3.2	Ex LLRmyc	Ex FLRmyc	ND
LOC_Os04g13090	Vignain precursor, putative (OsAM39)	4.3	Ex LLRmyc	Ex FLRmyc	ND
**The half-size abc transporters Gutjahr et al., 2012**					
LOC_Os09g23640	ABC-2 type transporter domain containing protein (*OsSTR1*–OsAM53)	No DEG	1.9	No DEG	No DEG
**H+-ATPASE Wang et al., 2014**					
LOC_Os03g01120	E1-E2 ATPase domain containing protein, expressed (OsAM43)	2.2	2.3	1.3	1.2

*Values correspond to Log2ratio. Ex LLRmyc, eclusively expressed in LLRmyc; Ex FLRmyc, exclusively expressed in FLRmyc; Ex LLRc, exclusively expressed in LLRc; Ex FLRc, exclusively expressed in FLRc; ND, not detected; no DEG: not differentially expressed gene*.

Considering the presymbiotic phase, it is worthwhile to note that the genes involved in strigolactone (SL) biosynthesis, such as carotenoid cleavage dioxygenase *(CCD)* 7 (*OsCCD7*), *OsCCD8a, OsCCD8b, OsCCD8c* and two cytochrome P450 genes (with high sequence similarity to the Arabidopsis SL biosynthesis gene MAX1, Cardoso et al., 2014) and genes which showed Lysin motif domain (LysM) (Table 1) were highly expressed in LLRmyc vs. FLRmyc. Moreover, the putative homolog of *Lotus japonicus* Lectin Nucleotide Phosphohydrolase (*LjLNP*), described by Roberts et al. (2013) as a Nod factor-binding protein required for AM symbiosis, showed higher expression in LLRmyc compared to FLRmyc (Table 1). As expected, the majority of the genes belonging to the CSSP were not differentially regulated between LLR and FLR in both conditions (myc and non-myc), with the exception of *OsNUP133* (Table 1).

We also observed that mycorrhization *per se* induced the up-regulation of some defense-responsive genes in LLRmyc compared to LLRc (Table S8); however a pathogenesis-related Bet v I family protein (a putative *OsPR10*–LOC_Os12g36840) and two jasmonic acid-induced protein Jacalin-related lectins (JRLs) (LOC_Os01g25280; LOC_Os06g07250) were strongly induced in FLRmyc compared to both LLRmyc and FLRc.

Interestingly, we observed that the transcripts of genes acting down-stream of the CSSP, such as those belonging to the GRAS-domain proteins complex (*DELLA/SLR1*, Required for Arbuscular Mycorrhization - *RAM1*; the putative homolog of Required for Arbuscule Development 1–*RAD1*) (Gobbato et al., 2012; Floss et al., 2013; Yu et al., 2014; Xue et al., 2015) and *RAM2*, considered crucial for hyphopodium formation, were barely expressed in FLRmyc compared to LLRmyc.

To investigate whether the absence of *RAM2* induction in FLR was related to insufficient production or release of cutin or related compounds, which in turn affects hyphopodia formation, we treated rice mycorrhizal plants with C16 cutin/suberin monomers and we assessed the presence of fungal hyphopodia on roots. The addition of the C16 monomers was not sufficient to compensate for *RAM2* repression: in fact, we did not observe either an induction of hyphopodia formation on FLR (data not shown) or an increase of AM colonization level on LLR (Figure S5).

Comparison of the previously published rice AM marker genes and our transcriptomic data revealed that six genes (*AM2, AM15, AM29, AM31, AM104*, and *AM129*) were not differentially regulated in LLRmyc vs. LLRc, while five genes (*AM10, AM25, AM38, AM42*, and *AM85*) were strongly up-regulated in LLRmyc, but their transcripts were also detected in LLRc (Table 1). In agreement with published data, eight AM marker genes (*AM1, AM20, AM34, AM11, AM14, AM18, AM24*, and *AM26*) were specifically expressed in LLRmyc vs. LLRc.

All the other genes regulated upon myc treatment but not previously described in the literature in rice, were considered putative new rice transcripts involved in AM symbiosis. Depending on their expression level, we clustered them in the following categories: (i) novel rice AM markers, which are specifically induced in LLRmyc vs. LLRc and not detected in FLR in both conditions; (ii) AM-responsive genes, which are more expressed in LLRmyc vs. FLRmyc and not detected in LLRc; (iii) AM-induced genes, which are strongly up-regulated in LLRmyc vs. LLRc and more expressed in LLRmyc vs. FLRmyc (Table 2).

**Table 2:** **List of the novel rice AM marker genes (specifically induced in LLRmyc vs. LLRc and not detected in FLR in both conditions), AM-responsive genes (specifically induced in LLRmyc vs. FLRmyc and not detected in LLRc), and AM-induced genes (strongly induced in LLRmyc vs. LLRc and in LLRmyc vs. FLRmyc)**.

	**MSU LOC_OS ID**	**MSU description**	**Log2ratio LLRmyc vs. FLRmyc**	**Log2ratio LLRc vs. FLRc**	**Log2ratio LLRmyc vs. LLRc**	**Log2ratio FLRmyc vs. FLRc**	***Medicago truncatula***	***Lotus japonicus***
Novel AM marker genes	LOC_Os01g26340	Dirigent, putative	Ex LLRmyc	ND	Ex LLRmyc	ND		
	LOC_Os01g36350	Cytochrome P450, putative, expressed (P450 71C4)	Ex LLRmyc	ND	4.7	ND		
	LOC_Os04g56360	Cysteine-rich receptor-like protein kinase 8 precursor, putative, expressed	Ex LLRmyc	ND	Ex LLRmyc	ND		Chr4.CM0244.1000.r2.m ▴
	LOC_Os11g28540	Expressed protein	Ex LLRmyc	ND	Ex LLRmyc	ND		
Novel AM responsive genes	LOC_Os03g57730	Uclacyanin-2 precursor, putative	4.8	ND	Ex LLRmyc	Ex FLRmyc		
	LOC_Os04g38300	Hypothetical protein	4.4	ND	Ex LLRmyc	Ex FLRmyc		
	LOC_Os01g58660	LTPL29—Protease inhibitor/seed storage/LTP family protein precursor, expressed	4.4	ND	Ex LLRmyc	Ex FLRmyc		
	LOC_Os06g44430	Protein kinase, putative	4.3	ND	Ex LLRmyc	Ex FLRmyc		
	LOC_Os10g34884	RIPER7—Ripening-related family protein precursor, expressed	4.0	ND	Ex LLRmyc	Ex FLRmyc		
	LOC_Os12g17540	Vignain precursor, putative	3.8	ND	Ex LLRmyc	Ex FLRmyc	TC100440 ▴	Chr3.LjB03G07.10.r2.a ▴
	LOC_Os03g57740	Plastocyanin-like domain containing protein, putative	3.8	ND	Ex LLRmyc	Ex FLRmyc		
	LOC_Os02g29020	Cupin domain containing protein	3.7	ND	Ex LLRmyc	Ex FLRmyc	TC103891 ▴	Chr6.CM0437.260.nc ▴
	LOC_Os12g41260	Protein kinase domain containing protein, expressed	3.7	ND	Ex LLRmyc	Ex FLRmyc	AL385435=	Chr2.CM0081.630.r2.m=
	LOC_Os06g37190	Conserved hypothetical protein	3.6	ND	Ex LLRmyc	Ex FLRmyc		
	LOC_Os04g21160	Triacylglycerol lipase 1 precursor, putative	3.6	ND	Ex LLRmyc	Ex FLRmyc	TC110731 ▴	Chr6.LjT47N10.60.r2.a ▴
	LOC_Os02g20140	Protein kinase domain containing protein	3.5	ND	Ex LLRmyc	ND		
	LOC_Os05g45954	AP2 domain containing protein, expressed	3.2	ND	Ex LLRmyc	Ex FLRmyc	TC112242 ▴	Chr2.CM0608.1100.r2.m ▴
	LOC_Os10g34770	RIPER4—Ripening-related family protein precursor, putative	3.2	ND	Ex LLRmyc	Ex FLRmyc		
	LOC_Os10g34902	RIPER9—Ripening-related family protein precursor, putative	3.1	ND	Ex LLRmyc	Ex FLRmyc		
	LOC_Os11g17790	Inhibitor I family protein, putative	3.1	ND	Ex LLRmyc	Ex FLRmyc		
	LOC_Os11g26140	Cysteine-rich receptor-like protein kinase 4 precursor, putative	3.0	ND	Ex LLRmyc	Ex FLRmyc	Medtr7g116650.1 ▴	
	LOC_Os01g67650	Gibberellin response modulator protein, putative	2.9	Ex FLRc	Ex LLRmyc	No Deg	Medtr4g104020.1 ▴	Chr4.CM1864.540.r2.m ▴
	LOC_Os10g28380	MTN26L3—MtN26 family protein precursor, putative	2.9	ND	Ex LLRmyc	Ex FLRmyc		
	LOC_Os01g58650	LTPL35—Protease inhibitor/seed storage/LTP family protein precursor, putative	2.9	ND	Ex LLRmyc	Ex FLRmyc		
	LOC_Os07g38050	Expressed protein	2.8	ND	Ex LLRmyc	Ex FLRmyc		
	LOC_Os05g35960	Conserved hypothetical protein	2.1	Ex FLRc	Ex LLRmyc	1.4		
	LOC_Os04g47390	GDSL-like lipase/acylhydrolase, putative	2.0	ND	Ex LLRmyc	Ex FLRmyc		
	LOC_Os04g58680	Core histone H2A/H2B/H3/H4, putative, expressed	1.8	Ex FLRc	Ex LLRmyc	1.2		
	LOC_Os10g22630	Expressed protein	No deg	ND	Ex LLRmyc	Ex FLRmyc		
Novel AM induced genes	LOC_Os06g20140	Aspartic proteinase nepenthesin-1 precursor, putative	3.1	Ex LLRc	9.5	Ex FLRmyc		
	LOC_Os12g06900	Citrate-binding protein precursor, putative	3.5	No DEG	8.2	3.3		
	LOC_Os02g17900	Glycosyl hydrolases family 16, putative, expressed	4.3	-3.54	7.9	no DEG		
	LOC_Os01g31760	Acyl-ACP thioesterase, putative	3.5	Ex LLRc	7.8	Ex FLRmyc	TC99271 ▴	Chr5.CM0328.70.r2.d ▴
	LOC_Os10g29650	Retrotransposon protein, putative, unclassified, expressed	No Deg	No Deg	7.7	7.1		
	LOC_Os01g72540	*OsCML23*—Calmodulin-related calcium sensor protein	4.2	Ex LLRc	7.6	Ex FLRmyc	TC102361 ▴	
	LOC_Os05g22300	Transferase family protein, putative	3.4	Ex LLRc	7.3	Ex FLRmyc		
	LOC_Os11g18320	Hypothetical protein	3.1	Ex LLRc	7.0	Ex FLRmyc		
	LOC_Os05g49790	DUF538 domain containing protein, putative, expressed	1.8	No DEG	7.0	5.1		Chr3.CM2103.230.r2.a ▴
	LOC_Os01g19750	Glycosyl hydrolase, putative, expressed	3.3	No DEG	6.9	5.4		Chr5.CM0456.330.nd ▴
	LOC_Os08g35750	Cupin domain containing protein, expressed	2.6	Ex LLRc	6.7	Ex FLRmyc		
	LOC_Os10g23130	Cytochrome P450 88A1, putative	2.6	Ex LLRc	6.6	Ex FLRmyc		
	LOC_Os03g29150	NAD dependent epimerase/dehydratase family protein, putative, expressed	1.8	no DEG	6.4	4.4		
	LOC_Os06g01580	LTPL127—Protease inhibitor/seed storage/LTP family protein precursor	2.1	No DEG	6.0	2.5		
	LOC_Os10g04730	TKL_IRAK_DUF26-la.6—DUF26 kinases have homology to DUF26 containing loci, expressed	4.5	No DEG	5.8	no DEG	CB895041=	Chr5.CM0200.1860.r2.d=
	LOC_Os09g15240	Carotenoid cleavage dioxygenase, putative, expressed	3.2	Ex LLRc	5.7	Ex FLRmyc		
	LOC_Os10g38690	Glutathione S-transferase, putative	3.3	No DEG	5.5	No DEG		
	LOC_Os01g73250	Abscisic stress-ripening, putative, expressed	1.3	Ex LLRc	4.1	Ex FLRmyc		LjSGA_018093.1 ▴

*Nucleotide sequences of these novel rice AM-transcripts were used for reciprocal BLAST in Medicago truncatula (http://mtgea.noble.org/v3/) and Lotus japonicus (http://ljgea.noble.org/v2/) gene expression atlas web servers. Triangles refer to up- (▴), down- (▾), or (=) not regulated during AM symbiosis. Ex LLRmyc, exclusively expressed in LLRmyc; Ex FLRmyc, exclusively expressed in FLRmyc; ND, not-detected; no DEG, not differentially expressed gene; Ex LLRc, exclusively expressed in LLRc; Ex FLRc, exclusively expressed in FLRc*.

Using these criteria, we identified four AM-marker genes: *Dirigent putative*, which is classified as a disease resistance-responsive gene; a Cytochrome P450 gene (P450 71C4); a receptor kinase gene *CRRLPK-8*, which shows similarity with *L. japonicus receptor-like protein kinase* strongly induced in mycorrhizal roots (Guether et al., 2009); and a gene encoding an “expressed protein with unknown function” (Table 2). We also identified 25 novel AM-responsive genes and 18 transcripts strongly up-regulated in LLRmyc vs. LLRc, that have not been described in the rice-AM fungi interaction so far. Among the AM-responsive genes, seven and six transcripts, respectively, showed similarity with *M. truncatula* and *L. japonicus* genes previously detected in AM roots (Table 2).

Two AM-responsive genes that encode lipid transfer protein (LTP) (*LTPL29, LTPL35*) were also highly regulated in LLRmyc vs. FLRmyc. Blilou et al. (2000) showed that another LTP (*LTPL11*) is regulated in rice root in response to AM colonization during hyphopodia formation and decreases at the onset of the intercellular colonization of the cortex. We also observed that genes belonging to the Ripening-related family protein precursor (RIPER) family (Table 2) were strongly induced in LLRmyc. Among these, *RIPER3* (*OsAM8*) was previously identified as a mycorrhiza-responsive rice gene (Güimil et al., 2005).

In addition, we found two novel receptor kinases showing an interesting gene expression profile. A serine/threonine-specific receptor protein kinase-like, resulted to be strongly induced in FLRmyc vs. FLRc and not detected in host root, while a receptor-like protein kinase (LOC_Os05g25430), showing high sequence similarity with the Feronia sequence of Arabidopsis (*OsRLK-FER*), was up-regulated in LLRmyc vs. LLRc and not detected in non-host root (Table 1).

Deeper analysis of the generated data set revealed that the presence of the AM fungus elicits the differential expression of a large number of genes, opening the question whether and how these DEGs overlap with the general changes illustrated in Figure 1. To face this question, we first focused on the well-known anatomical differences between the two root types.

### Root radial anatomy

In their detailed description of rice anatomy, Rebouillat et al. (2009) identified epidermis, exodermis, sclerenchyma, endodermis, and central cylinder tissues in transverse sections of the three root types of the Nipponbare cultivar; they also found that FLRs had no cortical cell layer. To test if this description could also be applied to the Selenio cultivar, we stained root sections for lignin and suberin/cutin, as cell wall markers to identify root tissues, and examined the sections by light and transmission electron microscopy.

**Figure 4:**
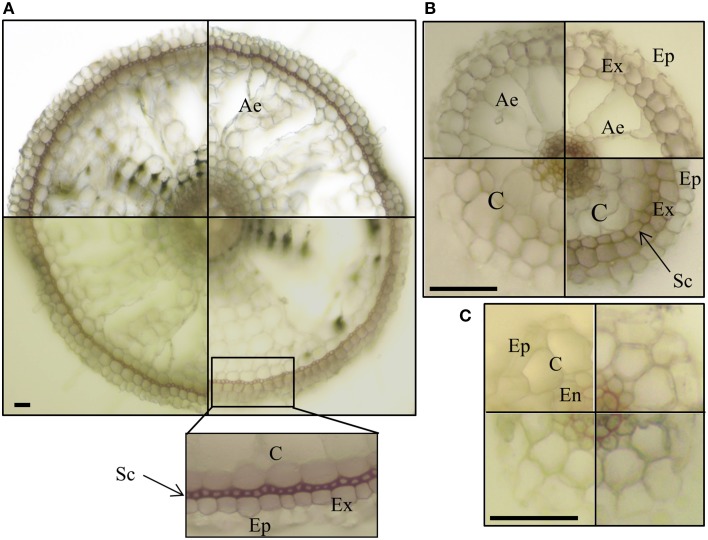
**Lignin staining of root sections**. Vibratome cross sections of crown roots–CR **(A)** large lateral root–LLR **(B)** and fine lateral roots–FLR **(C)** were stained with phloroglucinol–HCl. Presence of lignin was detected, as red color, in cell walls of xylem and endodermis Casparian bands in all the root types, and in cell walls of exodermis in CR and some LLR (**B**, right bottom box). Each box shows an independent root section. Ep, epidermis; Ex, exodermis; Sc, sclerenchyma; C, cortex; Ae, aerenchyma; En, endodermis. Bars correspond to 30 μm.

We observed the characteristic red color of lignin from phloroglucinol–HCl staining in the cell walls of xylem and endodermis Casparian bands in all root types (Figure 4). By contrast, only crown roots and a few LLR showed staining corresponding to the sclerenchyma layer (Figures 4A,B). This confirmed the presence of two types of LLR that differ in the presence (T-type) or the absence (L-type) of sclerenchyma (Kono et al., 1972). No lignified cells were detected in FLRc, suggesting that this root type in the Selenio cultivar does not develop exodermis and sclerenchyma layers, while a two layer-non-lignified cortex was consistently present (Figure 4C). No differences were detected in root-cross sections stained with the lipophilic dye Sudan 7B (data not shown) suggesting that suberin/cutin is not a relevant component of the cell walls of LLR and FLR.

**Figure 5:**
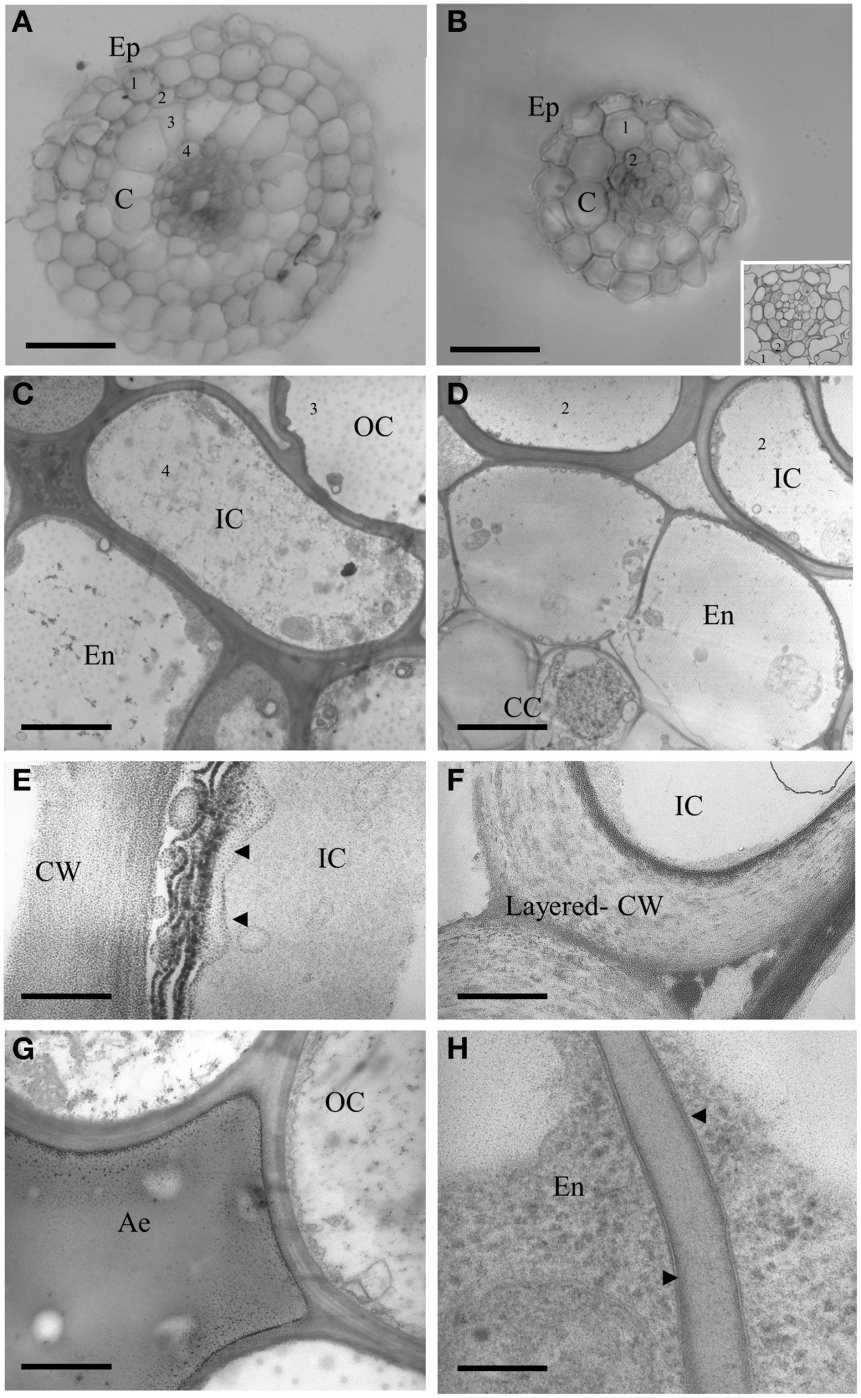
**Cross sections of LLR (left side) and FLR (right side) observed under optical and electron microscope**. Roots were embedded in agarose and vibratome sectioned **(A,B)** or embedded in resin and cut with an ultramicrome to obtain semi-thin (**B** inset) or ultra-thin **(C–H)** sections. Ultra-thin sections were treated with Thiery's polysaccharide stain. EP, Epidermis; C, Cortex; OC, Outer Cortical Cell layer; IC, Inner Cortical Cell layer; En, endodermis; CC, Central cylinder; CW, cell wall; Ae, aerechyma; 1-4, indicate cortical cell layers. Arrowheads indicate vesicles with a positive reaction to the Thiery's stain in **(E)** and thin suberin layer in **(H)**. Bars correspond to: 30 μm in **(A,B)**, 2.2 μm in **(C)**, 3.3 μm in **(D)**, 0.16 μm in **(E,H)**, 0.8 μm in **(F)**, 1.6 μm in **(G)**.

To get a deeper insight into the anatomy of the two root types, we also embedded LLR and FLR in resin, obtained semi- and ultra-thin sections and observed them by light and transmission electron microscopy. Vibratome sections (500 μm, Figures 5A,B) confirmed differences in radial anatomy, with four cortical layers in the LLR and only two in the FLR. The details of the central cylinder were better revealed in semi-thin sections (0.5 μm) showing a layer of roundish endodermal cells (Figure 5B, inset) surrounded by an inner cortical layer (layer 2). Ultrathin sections (0.05 μm) treated with Thiery's polysaccharide stain (Thiéry, 1967), to better detect cell wall organization, also revealed subtle differences. The LLR endodermal cells were rich in cytoplasm and in direct contact with the inner cortical cells (Figure 5C), where abundant vesicles with a positive reaction to the Thiery's stain lined the periplasmic area between the plasma membrane and the cell wall (Figure 5E), suggesting active polysaccharide secretion toward the multilayered wall. By contrast, in FLR, the endodermis did not show any cytoplasm and was in contact with a highly differentiated layer of cortical cells, which consisted of oval-shaped cells with a very thick, layered cell wall (Figure 5D). This cell wall strongly reacted with the silver grains of the Thiery reaction revealing a thin, multilayered organization, typical of fibrillar cellulose (Figure 5F). Lastly, both root types revealed thin Casparian bands with a very thin suberin layer localized exclusively in the central part of the radial endodermis walls (Figure 5H).

**Figure 6:**
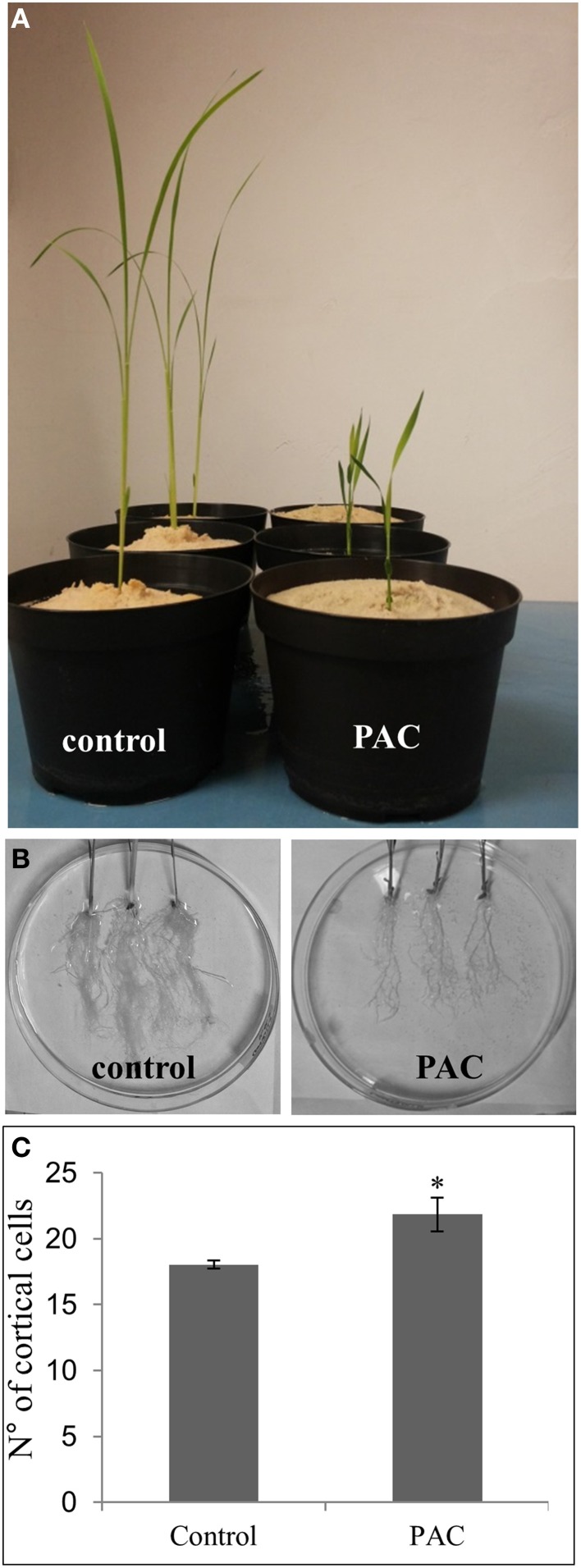
**Effect of paclobutrazol (PAC) treatment on rice plants**. The inhibitor of GA biosynthesis reduced growth of the aerial part **(A)** and of the root apparatus **(B)**, and increased the number of cortical cells in FLRc **(C)**. Error bars represent standard deviation. Asterisk indicates significant difference at *p* < 0.05, according to the One-Way ANOVA with Tukey's *post-hoc* test.

To better understand the molecular causes of the different numbers of cortical cell layers between LLR and FLR (Figure 5), we hypothesized that gibberellic acid (GA) may have a role. On one hand, GA is a key factor that affects asymmetric cell divisions in the ground tissue (Paquette and Benfey, 2005; Koizumi and Gallagher, 2013); on the other hand, genes related to GA metabolism and perception are differentially expressed between the two rice root types (Table S9). To test this hypothesis, we treated rice plants with paclobutrazol (PAC—an inhibitor of giberellin acid biosynthesis) and after 21 days of growth they showed the typical reduced internodal growth and root elongation (Figures 6A,B). Thirty vibratome cross sections of FLR from treated and untreated plants were examined under the microscope for ground tissue patterning. A statistically significant higher (*p* < 0.05) number of cortical cells was observed in FLR of plants treated with PAC than in control plants (Figure 6C). These data support the hypothesis that the different anatomy may be related to the regulation of plant phytohormones, and that in root, the anticlinal cell division is influenced by GA level.

Overall, these data demonstrate that FLR of the Selenio cultivar have two cortex layers without exodermal and sclerenchyma tissues. Transcriptomic data corroborated such morphological observations since genes involved in suberin and lignin biosynthesis were mainly induced in LLRc and not in FLRc (Table S9).

Lastly, morphological observations revealed that—notwithstanding the known anatomical differences—FLR possess indeed a cortical parenchyma, which makes them theoretically capable to host intracellular AM structures.

### Root phosphorus content

Since transcriptomic data revealed a consistent enrichment in nutrient transporters in FLR vs. LLR (Tables S9, S10), to validate whether a different nutritional uptake could be assigned to the two root types, we quantified the phosphorus contents of the two roots in plants grown in absence of the fungus. The P content of FLRc (2.10 ± 0.4 mg/g dry weight) was statistically significantly higher (*p* < 0.05) than LLRc (1.51 ± 0.2 mg/g dry weight). Thus, FLRc contained approximately 30% more P than LLRc. In the presence of the fungus, LLRmyc (1.81 ± 0.3 mg/g dry weight) showed higher P content than FLRmyc (1.42 ± 0.4 mg/g dry weight). Even though the increment was not statistically significant, the result suggests that in the presence of the fungus, LLR exploit the mycorrhizal phosphate uptake pathway, balancing the direct pathway by FLR.

## Discussion

AM fungi colonize plant roots by a series of spatio-temporal steps. After a chemical dialog between the two symbionts (Bonfante and Genre, 2015), the fungus reaches the root surface and forms the hyphopodium, from which a penetration hypha invades the rhizodermal cell layer. The intracellular hyphae rapidly develop into the plant cortex and form the arbuscule, which is the functional site where bidirectional nutrient exchange takes place between the host and the fungus.

The root apparatus of rice plants offers a powerful, unique tool to study the plant-AM fungus interaction, since it consists of both host and non-host roots, thus allowing their direct comparison in the same genetic background and also in the same individual plant. In our work, we took advantage of this peculiarity to investigate the determinants involved in AM core colonization. To this end, we first obtained a whole-transcriptome data set from LLR and FLR and examined their responses upon mycorrhizal colonization. Subsequently, we mined the data set for the expression profiles of genes involved in AM symbiosis, keeping in mind the main phases of fungal colonization and combining the results with anatomical and physiological evidences.

### Genes involved in presymbiotic recognition

As illustrated in many reviews (Bonfante and Requena, 2011; Gutjahr and Parniske, 2013; Schmitz and Harrison, 2014; Bonfante and Genre, 2015), plants dialog with AM fungi thanks to the release of signal molecules like strigolactones (SL), the activation of signaling pathway genes belonging to the CSSP, as well as the activation of defense related genes. On the one hand, our data from the RNA-seq experiment confirmed that the majority of the CSSP genes were not differentially regulated (Gutjahr et al., 2008). On the other hand, genes involved in SL biosynthesis and defense reactions were differentially expressed. In fact, consistent with the observation that LLR are susceptible to mycorrhization, we found an induction of genes involved in SL biosynthesis compared to FLR, in both control and mycorrhizal conditions (Table 1). By contrast, FLRmyc showed a higher expression of defense-response genes (Table 1). The accumulation of pathogenesis-related proteins represents a ubiquitous response to pathogen infection in plants (van Loon et al., 2006), but the up-regulation of genes involved in defense also occurs in response to mycorrhization (Campos-Soriano et al., 2010; Lopez-Raez et al., 2010). Such a host response is probably under the control of a finely tuned phytohormonal network, but experimental data are at the moment not conclusive (Lopez-Raez et al., 2010; Kloppholz et al., 2011; Plett et al., 2014). In our experiment, we observed that two jasmonic acid-induced JRLs were strongly induced in FLRmyc compared with both LLRmyc and FLRc. New roles are emerging for JRLs, which are a subgroup of proteins with one or more jacalin-like lectin domains. Interestingly, rice JRLs are associated with biotic or abiotic stimuli, such as salt stress or pathogen infection (Garcia et al., 1998; Zhang et al., 2000; Qin et al., 2003); however, their biological functions in plants are still poorly understood (Ma et al., 2010; Al Atalah et al., 2011; Xiang et al., 2011; Balsanelli et al., 2013).

Taken in the whole, the transcriptomic data show that the repression of genes involved in SL biosynthesis and the induction of defense genes might have a role in preventing AM fungal colonization in FLR. Otherwise, the induction of the defense responsive genes in FLRmyc might be a consequence of the mycorrhizal priming effect.

### Downstream of the common symbiotic signaling pathway

It was suggested that the formation of a large complex of GRAS-domain proteins (*DELLA/SLR1*, DELLA Interacting Protein 1—*DIP1, RAM1*, and *RAD1*) is a prerequisite for elicitation of nodulation or mycorrhization (Oldroyd, 2013).

In line with the results obtained in mycorrhizal *M. truncatula* roots (Floss et al., 2013) we observed a down-regulation of *DELLA* transcript in LLRmyc compared to LLRc. Although DELLA is involved in arbuscule formation through the repression of gibberellin signaling (Floss et al., 2013; Yu et al., 2014), *DELLA* expression is high during Pi-limiting conditions. Once arbuscules form, symbiotic Pi transport leads to an increase in Pi levels in the root, resulting in a decrease in *DELLA* transcript levels (Floss et al., 2013). Moreover, we found further repression of *DELLA/SLR1* and *OsRAM1* expression (Table 1) in FLRmyc vs. LLRmyc. A lower *DELLA/SLR1* mRNA abundance was also detected in FLRc vs. LLRc suggesting that this transcript regulation is probably related to the different morphophysiological features of the two root-types. By contrast, the lack of induction of *OsRAM1* prompts us to speculate that in FLRmyc the mycorrhizal signaling pathway is not activated (Gobbato et al., 2012).

Downstream of the complex of GRAS-domain proteins, the glycerol-3-phosphate acyl-transferase RAM2 functions in the production of cutin monomers and induces hyphopodium formation (Wang et al., 2012). Cutin monomers can guide and stimulate the initial approach of the AM fungus and hyphopodium formation which requires cutin monomers (Gobbato et al., 2012; Wang et al., 2012; Gobbato et al., 2013). Cutin mostly occurs in the aerial part of the plant, providing a hydrophobic surface that pathogens exploit in the early stages of the interaction. In general, roots do not contain cutin, but instead contain the related compound suberin. No difference in suberin/cutin deposition was observed, suggesting that they are not relevant components of the cell walls of LLR and FLR. Furthermore, the addition of C16 monomers upon AM fungus inoculation did not elicit hyphodopia formation on FLR, suggesting that the absence of fungal structures on FLR does not directly depend on the availability of cutin/suberin monomers.

### Intracellular phase: The cortex is necessary but not sufficient for AM symbiosis

Since intercellular hyphae and arbuscules require cortical cells, Gutjahr et al. (2009) proposed that the lack of cortex tissue in FLRs, as described in previous work (Rebouillat et al., 2009), can be a key factor for their inability to form arbuscules. However, FLR of the Selenio cultivar do have cortical layers, but are not susceptible to fungal colonization. Moreover, the lack of hyphopodia on the FLR surface implies other mechanisms, such as insufficient release of diffusible molecules and/or lack of a specific surface signal required for fungal attachment and hyphopodium induction.

LLR have more cortical layers than FLR. The higher expression of the DELLA/SLR1 and of the gene encoding the cytochrome P450 CYP714B1 (Magome et al., 2013) detected in LLRc compared with FLRc (Table S9), suggests that a repression of GA signaling may occur in LLRc, leading to multiple cortical layers. In fact, GA also acts in a partially overlapping pathway with the GRAS family transcription factors Short-Root (SHR) and Scarecrow (SCR) to regulate asymmetric cell divisions in the ground tissue (Paquette and Benfey, 2005; Cui et al., 2007; Koizumi and Gallagher, 2013). Consistent with previous studies (Paquette and Benfey, 2005; Koizumi and Gallagher, 2013), we observed significantly more cortical cells in FLR of plants treated with PAC (GA-inhibitor) (Figure 6C), suggesting that GA inhibition, at least in FLR, induces anticlinal cell division.

The role of GA in AM symbiosis is constantly evolving. Recent works demonstrated that the inhibition of GA biosynthesis or the suppression of GA signaling can strongly inhibit arbuscular mycorrhiza development in the host root (Floss et al., 2013; Foo et al., 2013; Takeda et al., 2015). The spatial–temporal regulation and the fine-tuning of the GA level is necessary to promote AM colonization. Considering our data, it is tempting to speculate that GA has pleiotropic effects, since it affects root anatomical traits and in turn potentially influences the symbiosis signaling pathway.

### Expression of rice AM marker genes

Previous studies on the transcriptome of rice mycorrhizal roots identified a group of genes exclusively induced by AM fungi (Güimil et al., 2005; Gutjahr et al., 2008). Interestingly, we found basal expression of all the AM marker genes in FLRmyc (Table 1). This could be attributed on the one hand to a systemic alteration in gene expression, as previously demonstrated in non-mycorrhizal halves of the root system (Pozo et al., 2002; Gutjahr et al., 2008), or on the other hand, to a specific molecular dialog between a non-host root and the AM fungus.

We also detected an up-regulation of *OsPT11* in FLRmyc vs. FLRc and a slight induction of *OsPT13* in FLRmyc vs. LLRmyc (Table 1); the expression of these two PT genes in a non-host root was surprising since they are strongly induced in AM symbiosis and involved in arbuscule formation (Paszkowski et al., 2002; Yang et al., 2012). The systemic expression in FLR of genes previously described as AM-marker genes suggests that in our system these genes might perform other functions. For instance, the AM marker *LjLTP4* (*L. japonicus* homolog of *OsPT11*) was detected in the apex of non-inoculated roots (Volpe et al., 2012), indicating that it may function in root meristem in an AM symbiosis-independent manner.

### FLR and AM fungi: Do they understand each other?

Differently from the microarray-based work of Gutjahr et al. (2015), that did not reveal transcriptomic differences between FLR and LLR, our RNA-seq analysis showed impressively different expression profiles in the two types of lateral roots, including changes in many relevant metabolic activities, from cell division to phytohormone balance (i.e., gibberellin). These findings provide some putative explanations to help us understand the mechanism that make FLR recalcitrant to AM fungal colonization. Considering the morphological and physiological results, it is tempting to speculate that the two cortical cell layers present in Selenio FLRs are not sufficient to support the formation of arbuscules. As an alternative hypothesis, FLR's efficient role in nutrient uptake leading to a consistently higher P flow, may repress signaling pathways that are influenced by Pi levels (Russo et al., 2013). Along these lines, the genes involved in strigolactone biosynthesis and in GA-signaling are less expressed in FLR compared to LLR.

The absence of fungal hyphopodia adhering to FLR provides excellent morphological support for the transcriptomic data. Since the expression analysis revealed that transcripts of genes involved in the AM presymbiotic phase are almost absent in FLRmyc, we suggest that fungal hyphopodia directly or indirectly require such transcripts for their morphogenesis. The missed induction of the “symbiotic toolkit” genes in FLR is a unique biological trait, since non-host plant genomes generally lack these genes (Delaux et al., 2013). By contrast, a specific transcriptional program is switched off in FLR, leading to plant-fungus incompatibility.

Coming back to our initial hypotheses, we can conclude that a strong regulation of gene expression leads to the heterogeneity of lateral roots of rice, in line with the different transcriptional profiles of CR vs. lateral roots detected by Gutjahr et al. (2015). As a consequence, the two root types investigated here have different functions and anatomy, with FLR as the most competent for successful mineral nutrition. However, in contrast to one of our hypotheses, the different anatomy does not seem to have a major effect on AM colonization, while the deep differential regulation of genes involved in signaling could impair the initial steps of colonization.

On the basis of this work, and thanks to its peculiar root system, we propose rice as a useful instrument to pave the way to discover new molecular determinants underlying successful and unsuccessful root colonization by AM fungi.

## Author contributions

VF and MV carried out the majority of the experiments and wrote the manuscript. CB contributed to the RNAseq library preparation. AF carried out the electron microscopy experiments. P.Bag. performed the bioinformatics data analysis. PB coordinated the project, designed the experiments, and wrote the manuscript. All the authors read and approved the final manuscript.

### Conflict of interest statement

The authors declare that the research was conducted in the absence of any commercial or financial relationships that could be construed as a potential conflict of interest.
