# Supplementary figures and images for: Host and non-host roots in rice: cellular and molecular approaches reveal differential responses to arbuscular mycorrhizal fungi
Source: Front Plant Sci. 2015 Aug 13;6:636. doi: 10.3389/fpls.2015.00636 (PMC4534827; doi:10.3389/fpls.2015.00636)

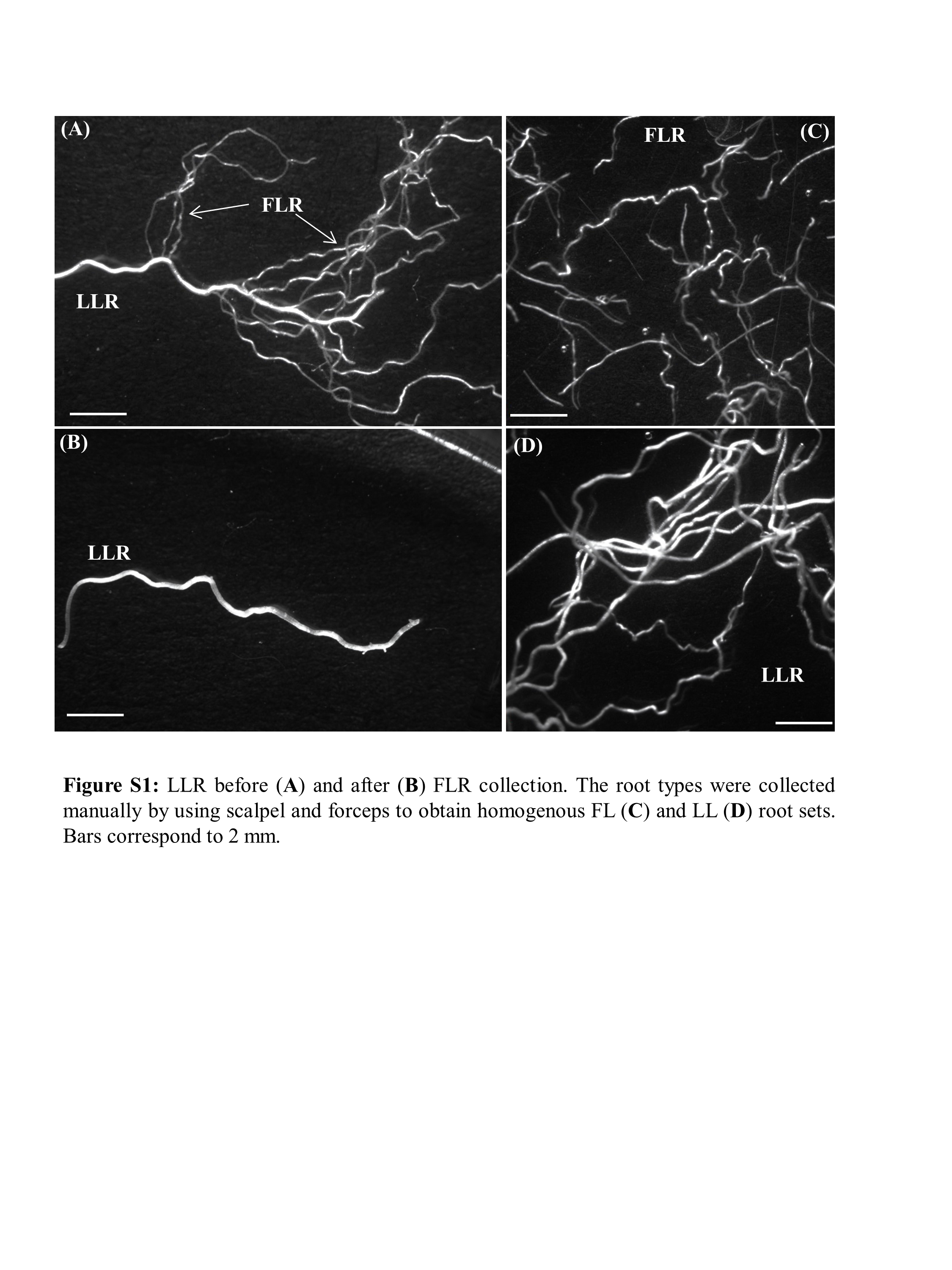

Supplement: Supplementary file 11 [file Image1.TIF]

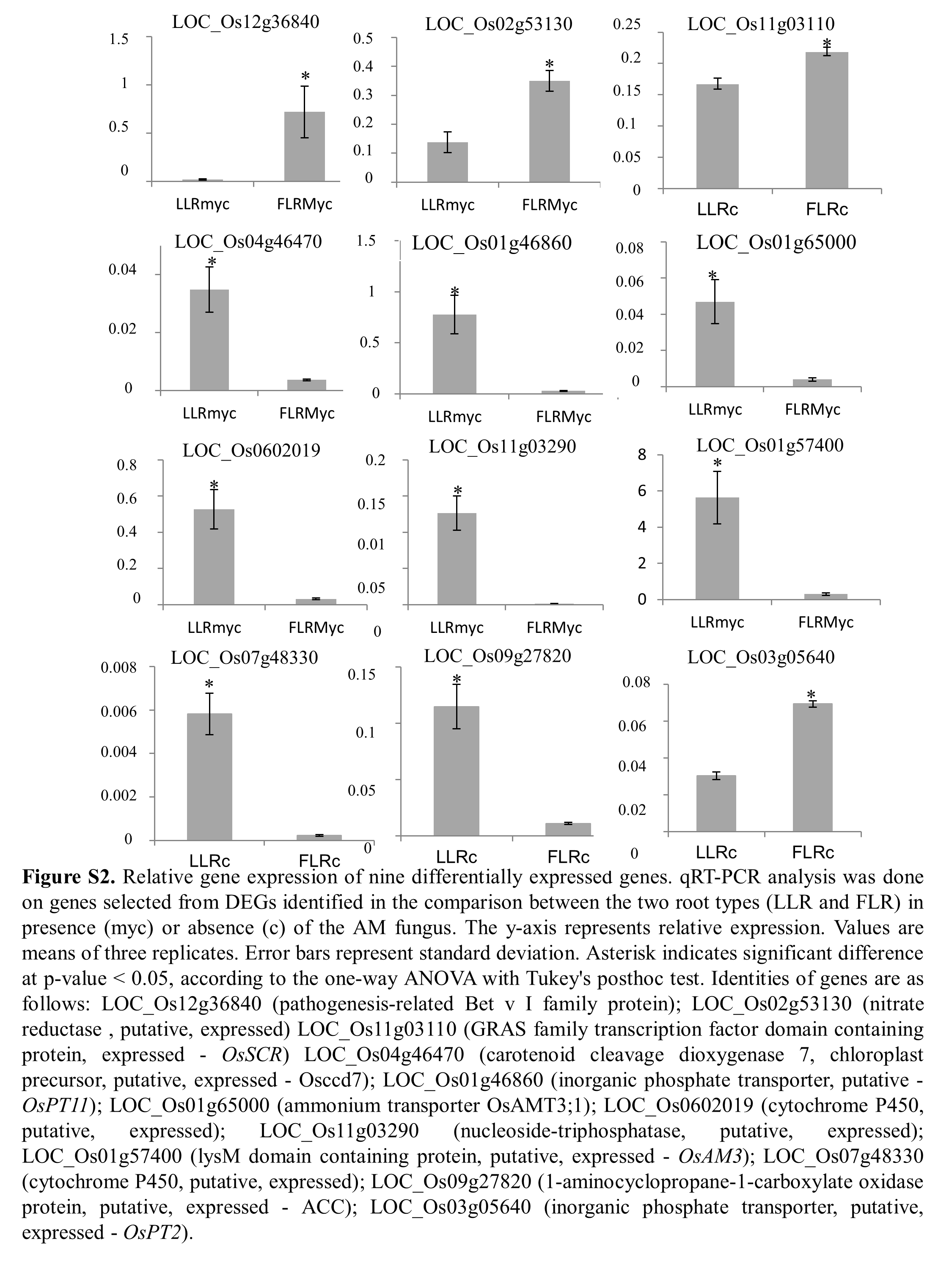

Supplement: Supplementary file 12 [file Image2.TIF]

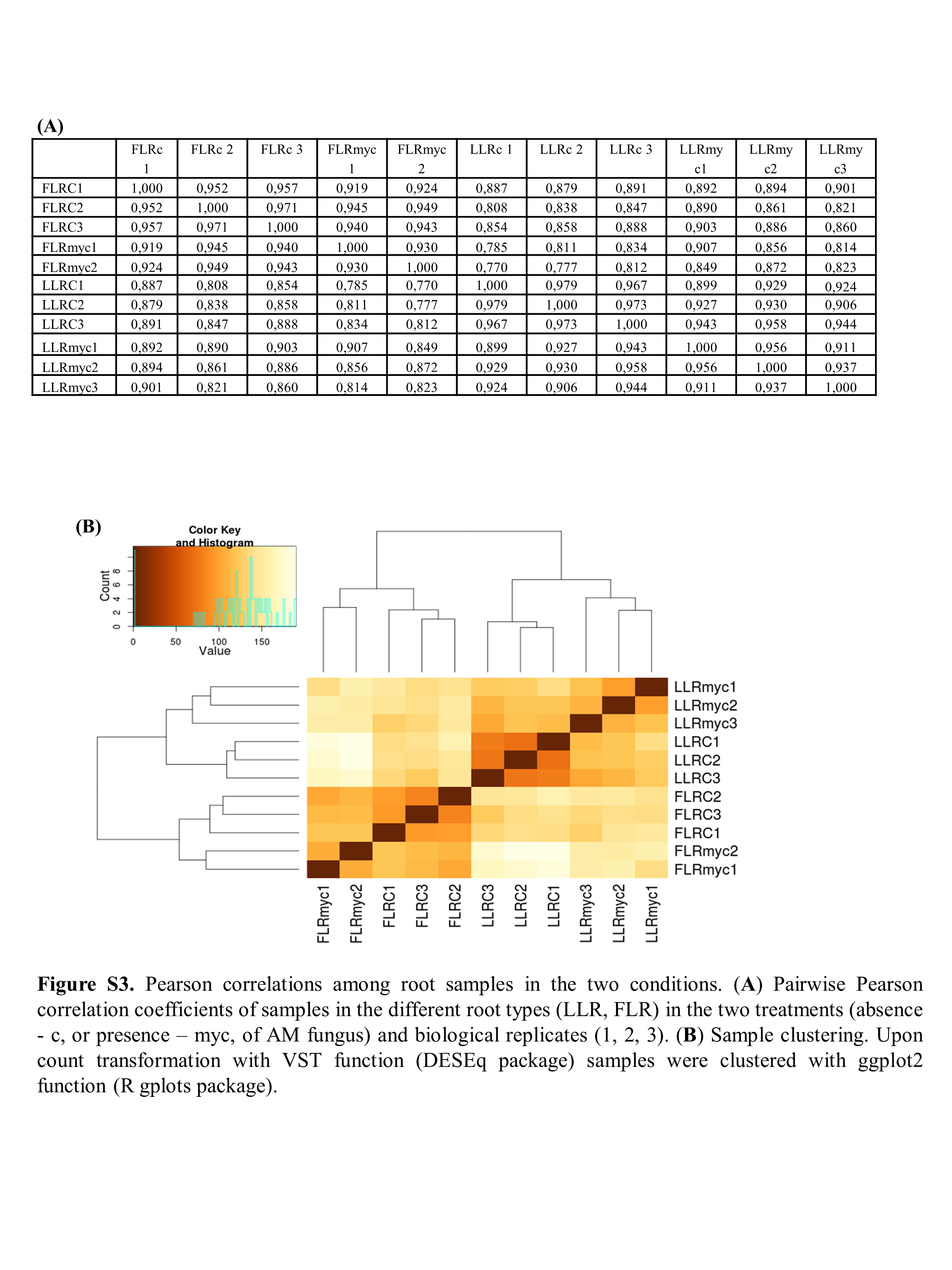

Supplement: Supplementary file 13 [file Image3.TIF]

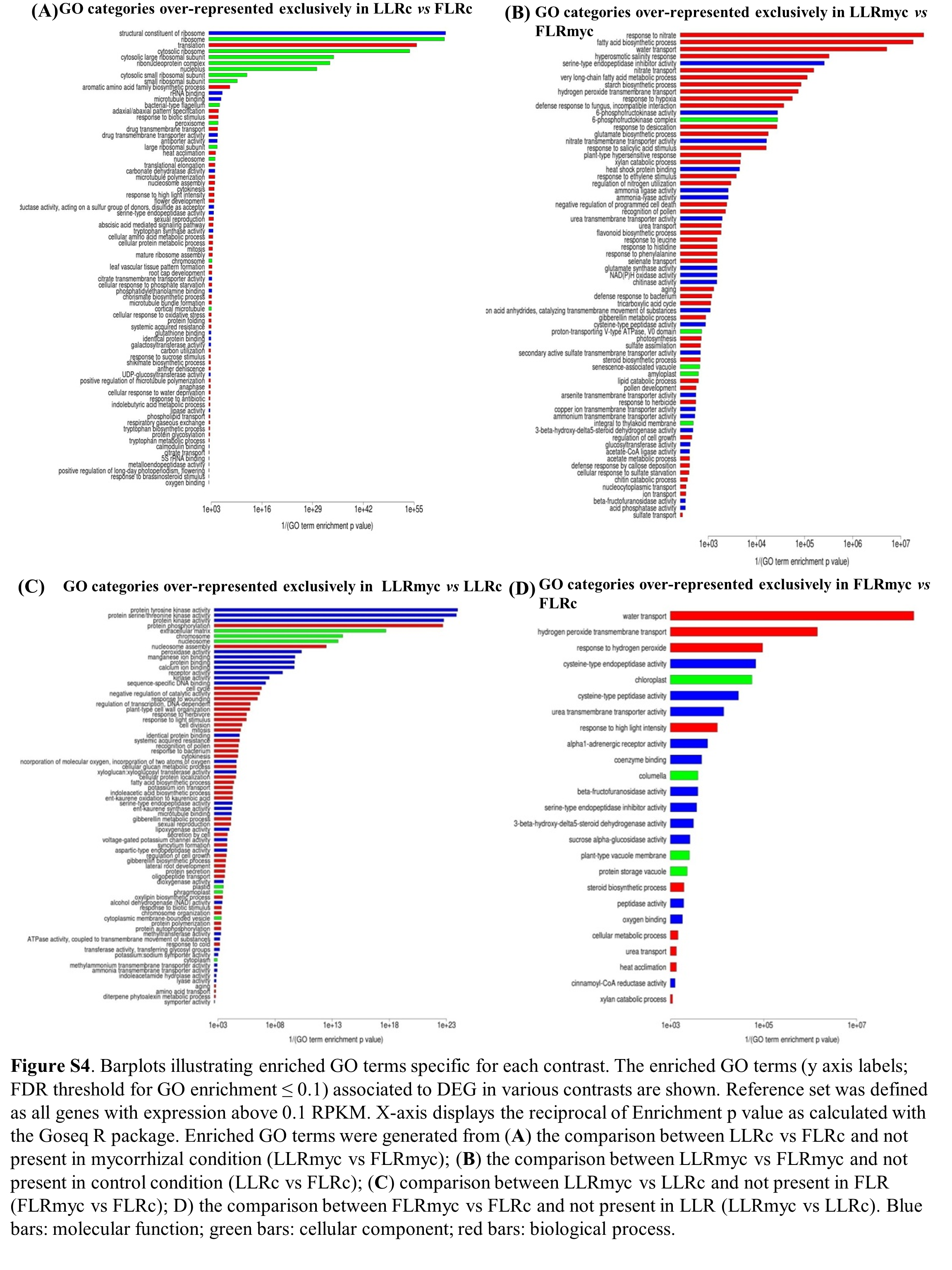

Supplement: Supplementary file 14 [file Image4.TIF]

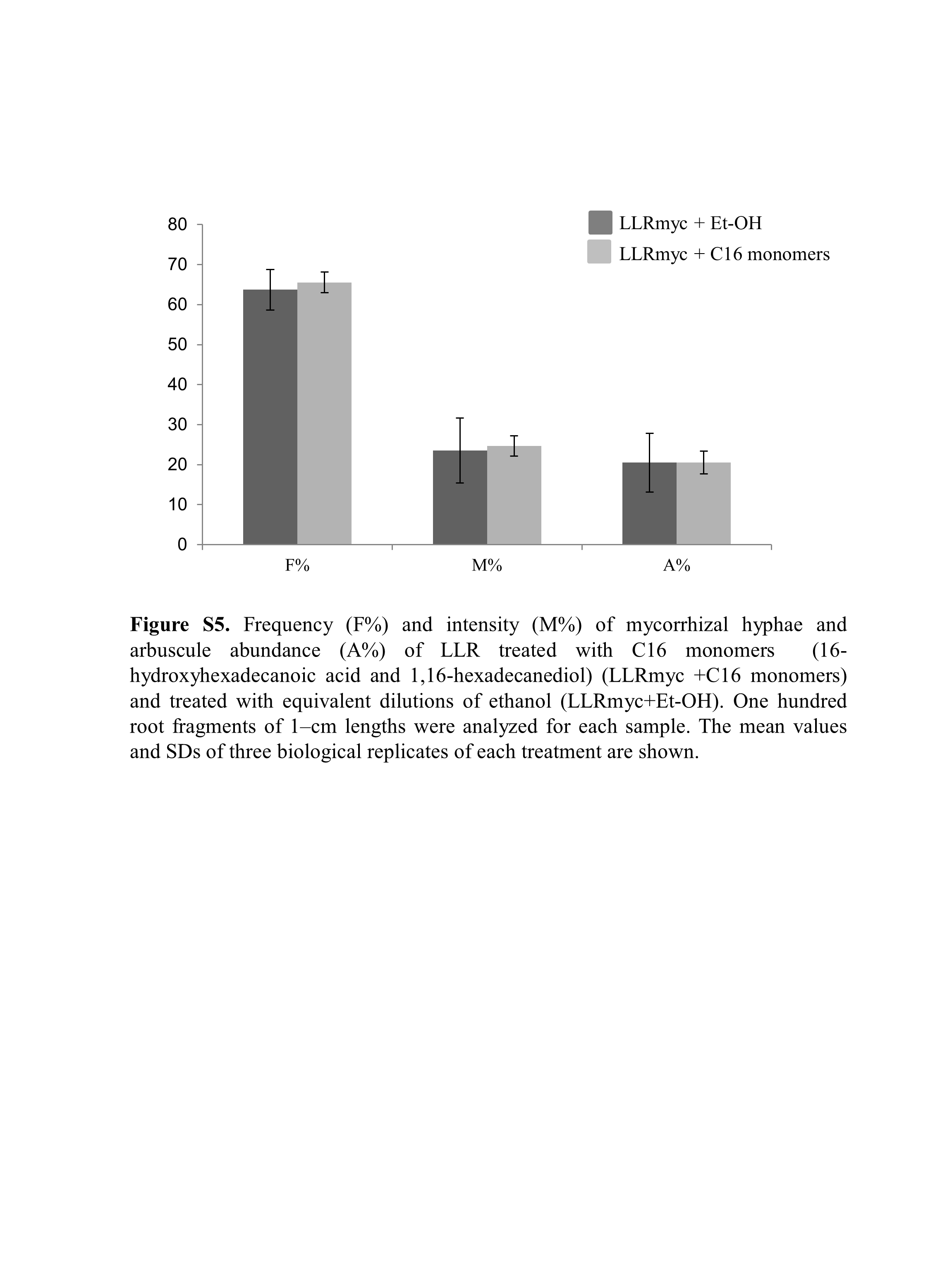

Supplement: Supplementary file 15 [file Image5.TIF]
